# Design and intelligent control of a low-cost, sEMG-driven, 3D-printed robotic hand prosthesis: A three-tier benchmarking framework with novel robustness metrics

**DOI:** 10.2478/joeb-2026-0016

**Published:** 2026-10-27

**Authors:** Anagha Shinde, Virendra Shete, Ninad Mehendale

**Affiliations:** School of Eng. and Science, MIT ADT Campus, MIT Art, Design and Technol. University, Loni Kalbhor, Pune 412201, Maharashtra, India; K. J. Somaiya School of Engineering, Somaiya Vidyavihar University, Mumbai 400077, India

**Keywords:** Surface electromyography, myoelectric prosthesis, MyoWare sensor, Random Forest, Deep learning, 3D-printed prosthetic hand, Low-cost assistive technology, Arduino, EMG classification, Ensemble learning, Robustness Degradation Index

## Abstract

Upper-limb amputation and neuromuscular disorders impose functional limitations on millions of people worldwide. Commercially available myoelectric prostheses, controlled by surface electromyography (sEMG) signals from residual limb muscles, cost approximately USD 75,000–100,000 (about INR 62–83 lakh), limiting accessibility in resource-limited settings. We present a 3D-printed, tendon-driven research hand prototype with a component cost of USD 122–232 (about INR 10,100–19,300), built around a single MyoWare 1.0 sensor and an Arduino Nano, with gesture classification performed on a tethered host PC.

Five classifiers—Random Forest (RF), Support Vector Machine (SVM), K-Nearest Neighbors (KNN), one-dimensional Convolutional Neural Network (1D CNN), and Long Short-Term Memory (LSTM)—were benchmarked across synthetic, Ninapro DB5, and live recordings of three gestures from ten able-bodied participants. Under subject-independent Leave-One-Subject-Out (LOSO) cross-validation, RF achieved the highest live accuracy (68.7%). Friedman and Nemenyi testing showed that RF was significantly better than SVM, KNN and LSTM (*p* < 0.05), while its advantage over the 1D CNN (60.7%) was not significant. Permutation importance identified waveform length, RMS and MAV as the most informative features.

We further propose two metrics: the Robustness Degradation Index (RDI), which quantifies the synthetic-to-real performance gap and was lowest for RF (43.7%), and a Cost–Performance Index (CPI), which was 13.77 for the prototype. The hand achieved an end-to-end latency of 285 ms and reliably held objects of up to 200 g. These results demonstrate well-above-chance three-class control with minimal hardware in able-bodied users and motivate evaluation with trans-radial amputees.

## Introduction

### Clinical and societal context

The World Health Organization (WHO) estimates that approximately 40 million people worldwide require prosthetic or orthotic services, yet only 5–15% of those in need have access to appropriate devices, particularly in developing nations [1]. Separately, an estimated three million people worldwide require a prosthetic arm [2]. Upper-limb loss, whether traumatic or congenital, profoundly impacts quality of life, functional independence, and psychosocial well-being [3]. Myoelectric prostheses—devices controlled by electrical signals generated during voluntary muscle contraction—represent the most physiologically intuitive control paradigm for artificial hands [4]. However, state-of-the-art commercial myoelectric systems, such as the Otto Bock Michelangelo Hand (approximately $75 000) and the DEKA LUKE Arm (approximately $100 000), remain financially inaccessible to the vast majority of potential users globally [5, 6].

This economic barrier has catalyzed a growing research interest in low-cost, open-source prosthetic systems that leverage affordable sensors, consumer-grade microcontrollers, and additive manufacturing (3D printing) technologies [7,8]. Surface electromyography (sEMG), which non-invasively measures the superposition of motor unit action potentials at the skin surface, serves as the primary biosignal modality for such systems [9]. The MyoWare Muscle Sensor (Advancer Technologies, USA), priced at approximately $38, has emerged as a popular low-cost sEMG acquisition platform in the maker and research communities due to its integrated instrumentation amplifier, envelope detection circuitry, and Arduino-compatible analog output [10].

### Research gap and motivation

Despite the proliferation of studies using low-cost sEMG sensors and machine learning for prosthetic control, several critical gaps persist in the literature.

**Gap 1: Over-reliance on synthetic or benchmark datasets.** The majority of published studies report classification accuracies exceeding 95 % on carefully curated, noise-free, or synthetically generated datasets [11, 12]. However, these results rarely translate to real-world deployment scenarios where electrode impedance variations, skin moisture changes, muscle fatigue, electrode displacement, and cross-talk introduce substantial signal degradation [13, 14]. Very few studies provide benchmarking on live-acquired, real-world sEMG signals alongside synthetic baselines, making it difficult to assess the practical viability of proposed systems.

**Gap 2: Limited multi-classifier comparison on single-channel systems.** While multi-channel sEMG systems (8–16 channels) using devices such as the Myo Armband or high-density electrode arrays have been extensively studied [15, 16], single-channel systems using sensors like the MyoWare have received comparatively less rigorous algorithmic attention. Single-channel systems are inherently more challenging due to reduced spatial information, yet they represent the most accessible and affordable option for resource-constrained settings. Comprehensive head-to-head comparisons of classical machine learning versus deep learning classifiers on single-channel, real-world sEMG data remain scarce.

**Gap 3: Absence of integrated cost–performance analysis.** Most studies focus exclusively on classification accuracy without contextualizing performance within the total system cost. For a prosthetic system to be genuinely accessible, both the algorithmic performance and the economic feasibility must be jointly evaluated and reported [17].

**Gap 4: Lack of standardised robustness metrics.** The sEMG literature lacks standardised metrics to quantify how much classification performance degrades when transitioning from controlled laboratory conditions to real-world deployment. Without such metrics, meaningful cross-study comparisons remain impossible, and the practical readiness of proposed systems cannot be objectively assessed.

**Gap 5: Lack of reproducibility and open-architecture reporting.** Many studies omit critical hardware details (baud rates, sampling protocols, electrode placement specifics) that would enable independent replication, hindering the cumulative progress of the field [18].

### Novel contributions of this study

This paper addresses the aforementioned gaps through the following original contributions.

**C1 Three-tier dataset benchmarking framework.** We introduce a systematic evaluation methodology comparing five classifiers across three progressively challenging data conditions—synthetic, online benchmark, and real-world live—using sampling-rate-appropriate preprocessing and common evaluation metrics. This framework explicitly quantifies the “reality gap” in sEMG classification, providing the research community with realistic performance expectations for low-cost systems.
**C2 Novel robustness and cost–performance metrics.** We propose two new evaluation metrics: the Robustness Degradation Index (RDI), which quantifies the fraction of above-chance accuracy lost when transitioning from synthetic to real-world conditions; and the Cost–Performance Index (CPI), which jointly evaluates classification agreement and economic cost. These indices complement conventional classification metrics.
**C3 Demonstration of ensemble learning performance on real-world single-channel sEMG.** RF significantly outperformed SVM, KNN and LSTM and achieved a higher mean accuracy than the 1D CNN. The RF–1D CNN rank difference of 1.2 did not exceed the Nemenyi critical difference of 1.929 at *α* = 0.05. These findings are restricted to the five classifiers, datasets and ten able-bodied participants evaluated here.
**C4 Complete cost–performance characterisation of an ultra-low-cost system.** We present a fully functional, 3D-printed myoelectric hand prosthesis system with component costs of approximately USD 122–232, a transparent component-level cost breakdown, and end-to-end performance evaluation from signal acquisition to mechanical actuation.
**C5 Reproducible, open-architecture system design.** All hardware specifications, software parameters, electrode placement protocols, and data collection procedures are documented in sufficient detail to enable independent replication.

### Paper organization

The remainder of this paper is organized as follows. Section Related work reviews related work. Section Materials and methods describes the materials and methods, including hardware design, data acquisition protocol, signal processing, feature extraction, classifier architectures, and the proposed novel metrics. Section Results presents experimental results. Section Discussion provides discussion and analysis. Section Conclusion and future work concludes with limitations and future directions.

## Related work

### Low-cost myoelectric prostheses

The convergence of affordable sEMG sensors, open-source microcontrollers, and desktop 3D printing has enabled a new generation of low-cost prosthetic research. Gusman et al. [19] developed an EMG-controlled prosthetic hand using the MyoWare sensor and Arduino Uno, achieving two-class binary open/close control using simple threshold processing rather than machine learning classification.

The e-NABLE community and projects such as the In-Moov robotic hand [20] have demonstrated the feasibility of 3D-printed prosthetic hands at costs below $500, but these systems typically employ simple on/off EMG control without multiclass pattern recognition capabilities. Tavakoli et al. [21] developed a low-cost myoelectric prosthesis using two EMG channels and an SVM classifier, achieving 85 % accuracy on controlled laboratory data; however, real-world performance was not evaluated and the system cost was not explicitly reported.

### sEMG classification approaches

The sEMG classification literature can be broadly categorised into classical machine learning and deep learning approaches. Hudgins et al. [22] established the foundational time-domain feature set—mean absolute value, waveform length, zero crossings, and slope sign changes—for myoelectric pattern recognition. Subsequent work demonstrated the effectiveness of SVM [23], LDA [24], KNN [25], and Random Forest [26] classifiers on these features, with Phinyomark et al. [27] identifying root mean square, mean absolute value, and waveform length as the most robust features across varying conditions.

On the deep learning side, Atzori et al. [28] applied convolutional neural networks (CNNs) to the Ninapro database, demonstrating that discriminative features could be learned directly from raw sEMG signals. Côté-Allard et al. [15] achieved state-of-the-art results using transfer learning with CNNs for inter-subject classification, while Simão et al. [29] applied LSTM networks to exploit temporal dependencies in muscle activation patterns. Importantly, these studies predominantly used multi-channel, research-grade sEMG systems costing $5 000–$50 000, making direct comparison with single-channel, low-cost configurations problematic.

### The synthetic-to-real performance gap

A critical but under-addressed issue in sEMG research is the dramatic performance degradation when classifiers trained on clean or synthetic data are deployed on real-world signals. Scheme and Englehart [13] documented accuracy drops of 20–40 percentage points between laboratory and practical conditions, with Campbell et al. [14] identifying electrode shift, muscle fatigue, limb position variation, and inter-session variability as primary contributors. Despite these well-known challenges, few studies explicitly quantify this gap by evaluating identical classifiers across synthetic, benchmark, and live data conditions within a single study, as we do here.

### Positioning of the present study

Table 1 summarises the positioning of the present study relative to key prior works.

**Table 1:** Comparison of the present study with selected prior works on low-cost sEMG prosthetic systems. NR = Not Reported.

**Study**	**Sensor**	**Ch.**	**Gest.**	**Classifiers**	**Real Data**	**Cost**
Gusman et al. [19]	MyoWare	1	2	Threshold	No	NR
Tavakoli et al. [21]	Custom	2	4	SVM	No	NR
Atzori et al. [28]	Otto Bock	12	50	CNN	No	NR
Côté-Allard et al. [15]	Myo Armband	8	7	CNN+TL	Partial	NR
Prakash et al. [30]	FMG (pressure)	1	6	Fuzzy control	Yes	NR
Sravya et al. [31]	MyoWare	1	3	SVM	No	NR
**Present Study**	**MyoWare 1.0**	**1**	**3**	**RF, SVM, KNN, 1D CNN, LSTM**	**Yes**	**$232**

Taken together, the reviewed studies commonly rely on multi-channel or research-grade acquisition, report benchmark or subject-dependent results, and provide limited comparisons of classical and deep learning methods under shared hardware constraints. Reproducible release of hardware, firmware and analysis code is also inconsistent. These limitations motivate the present single-channel feasibility study across synthetic, public benchmark and live multi-subject recordings, with subject-independent evaluation of the recorded datasets.

## Materials and methods

### System architecture overview

The proposed system comprises four functional subsystems: (i) sEMG signal acquisition, (ii) signal preprocessing and feature extraction, (iii) gesture classification, and (iv) mechanical actuation of the 3D-printed hand. The overall system architecture is depicted in Fig. 1.

**Figure 1:**
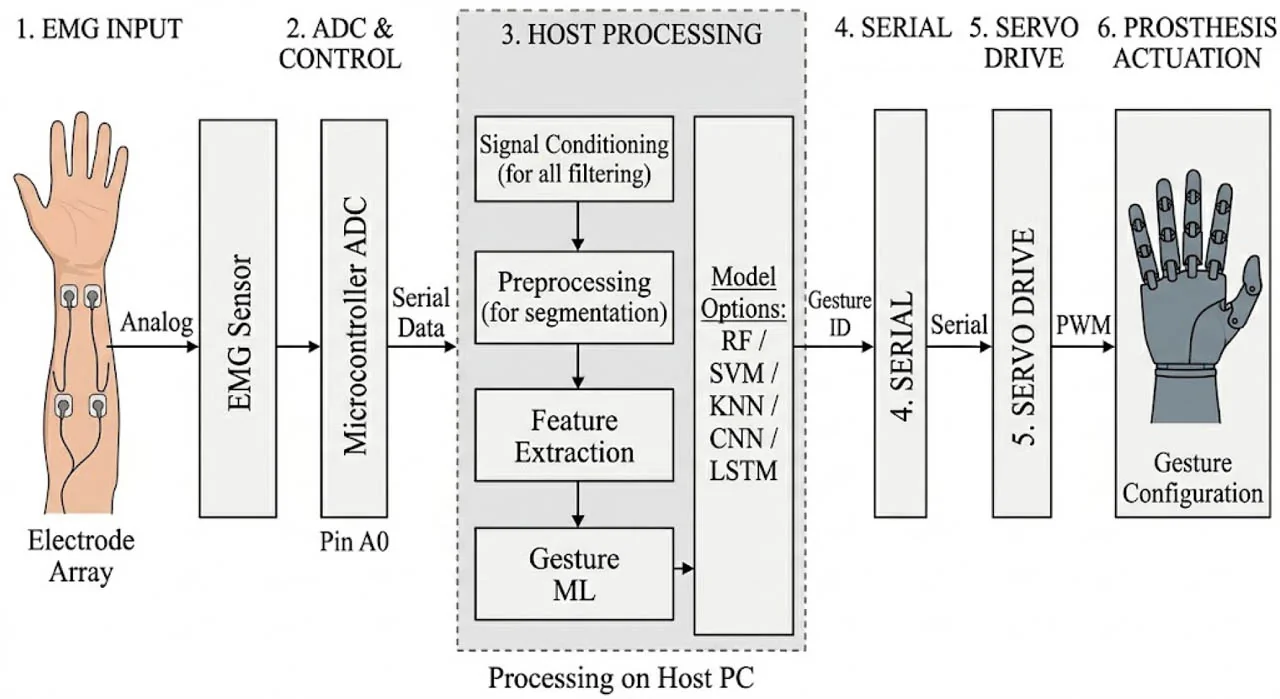
End-to-end system architecture of the proposed low-cost, sEMG-controlled, 3D-printed prosthetic hand. The dashed box denotes processing performed on a host PC; all other blocks are embedded hardware.

### Hardware components and cost analysis

#### MyoWare 1.0 muscle sensor

The MyoWare 1.0 (Advancer Technologies) is a single-channel analog EMG module with a differential electrode input, reference electrode, adjustable gain, and RAW and rectified/integrated SIG outputs. It operates from 2.9–5.7 V DC and uses MID, END and REF Ag/AgCl electrodes. In this study the amplified RAW output was connected to Nano A0, since it preserves the oscillatory waveform needed for time- and frequency-domain features; RAW is a single-ended signal biased at half the sensor supply voltage.

A single sensor was placed over the flexor digitorum superficialis (FDS), the principal extrinsic finger flexor, to evaluate the minimum-hardware configuration. The consequences of single-site sensing for gestures driven by other muscles are examined in Section Gesture-specific analysis.

#### Arduino Nano microcontroller

The Arduino Nano (Arduino S.r.l., Monza, Italy) was selected as the microcontroller platform owing to its compact form factor (18 × 45 mm), low cost (approximately $5 for compatible clones), and adequate computational resources for analog-to-digital conversion and servo control. The board is built around the ATmega328P 8-bit AVR core running at 16 MHz, with 32 kB of flash memory and 2 kB of SRAM. It provides 14 digital I/O pins (6 PWM-capable) and eight 10-bit analog input pins (0–1023 resolution), i.e. 22 I/O pins in total, and configurable UART communication (operated at 115 200 bps in this study).

The Arduino Nano served dual roles: acquiring the analog sEMG signal from the MyoWare sensor via its ADC and transmitting digitised samples to a host PC over USB serial at 115 200 baud for host-PC classification; and, in the reverse path, receiving classification results from the PC and generating PWM signals to drive the servo motors.

#### Servo motors and mechanical actuation

Five SG90 micro servo motors (Tower Pro, Taiwan) were used to actuate the five digits of the 3D-printed hand through a tendon-cable mechanism. Each SG90 provides 1.8 kg·cm of torque at 4.8 V, completes 60° of travel in 0.1 s, spans a rotation range of 0–180°, and weighs 9 g. Nylon monofilament fishing line (0.5 mm diameter) served as artificial tendons, routed through PTFE tubing channels within the 3D-printed hand structure. Each servo was mapped to one finger, with the thumb servo mounted at a 45° offset to enable oppositional grip.

#### 3D-printed hand

The prosthetic hand was fabricated using a Creality Ender-3 V2 fused deposition modelling (FDM) 3D printer with PLA (polylactic acid) filament (1.75 mm diameter). The hand design was adapted from the open-source In-Moov project [20] with modifications for tendon routing and servo mounting brackets. Print parameters were: 0.2 mm layer height, 30% gyroid infill, nozzle temperature 200 °C, bed temperature 60 °C, total print time approximately 28 h, and material consumption approximately 350 g of PLA. Finger joints are pin pivots connecting rigid PLA phalanx segments. The servo-driven nylon tendons run through PTFE tubing and attach to the distal finger segments. Extension is passive: a spring-loaded return bobbin attached to each tendon maintains tension and restores the open posture as the servo releases. The pivot pins are mild steel (12 mm diameter), and a 46320 return spring is used.

#### System cost breakdown

Table 2 lists representative unit prices for all components, including the servo power supply (Section Power distribution and load management): the 2S lithium-ion pack, LM2596 buck module and decoupling capacitors add USD 14.00, giving an itemized total of USD 132.50 (about INR 11,000). Depending on sourcing, the total component cost ranges from approximately USD 122 (optimized sourcing) to USD 232 (full retail); the upper value is used for the CPI as a conservative cost basis. All conversions use a fixed reporting rate of 1 USD = 83 INR: USD 122–232 corresponds to approximately INR 10,100–19,300, and USD 75,000–100,000 to INR 62–83 lakh.

**Table 2:** Itemized component costs at representative unit prices. Depending on sourcing, the total system cost ranges from approximately USD 122 (optimized sourcing) to USD 232 (full retail).

Component	Qty	Unit (USD)	Total (USD)
MyoWare 1.0	1	37.95	37.95
Arduino Nano compatible clone	1	4.50	4.50
SG90 servo	5	2.50	12.50
PLA filament (1 kg spool)	1	20.00	20.00
Ag/AgCl electrodes (50 pack)	1	8.00	8.00
Breadboard, wires and connectors	1 set	6.00	6.00
Nylon tendon line	1	4.00	4.00
PTFE tubing (1 m)	2	2.50	5.00
5 V USB power bank	1	8.00	8.00
Printing electricity	–	–	2.55
Miscellaneous solder and screws	–	–	10.00
2S lithium-ion pack, 7.4 V, 2000 mAh	1	11.50	11.50
LM2596 5 V buck module	1	1.80	1.80
1000 *μ*F and 100 nF capacitors	1 set	0.70	0.70

Itemized total			132.50

Retail prices of commercial devices also cover multi-channel sensing, custom socket fitting, clinical training and support, certification, warranty and durability testing. The cost comparison therefore indicates the scale of the hardware-cost opportunity rather than a like-for-like product comparison.

#### Power distribution and load management

The Nano and MyoWare are supplied from a 5 V USB source. The Nano 5V pin supplies MyoWare +*V_s_*, and RAW connects to A0. The five servo signal inputs connect to D3–D7. A separate 2S lithium-ion pack (7.4 V nominal, 2000 mAh) feeds an LM2596 buck module set to 5.0 V, with a nominal 3 A rating, supplying the servo positive terminals directly (Fig. 2). VIN is unused, and servo current does not pass through the Nano onboard regulator. The positive rails remain separate, with return paths meeting at a single star-ground point. A 1000 *μ*F low-ESR electrolytic and a parallel 100 nF ceramic capacitor are placed across the servo rail at the load. This routing reduces shared-return interference between the actuation and sensing circuits.

**Figure 2:**
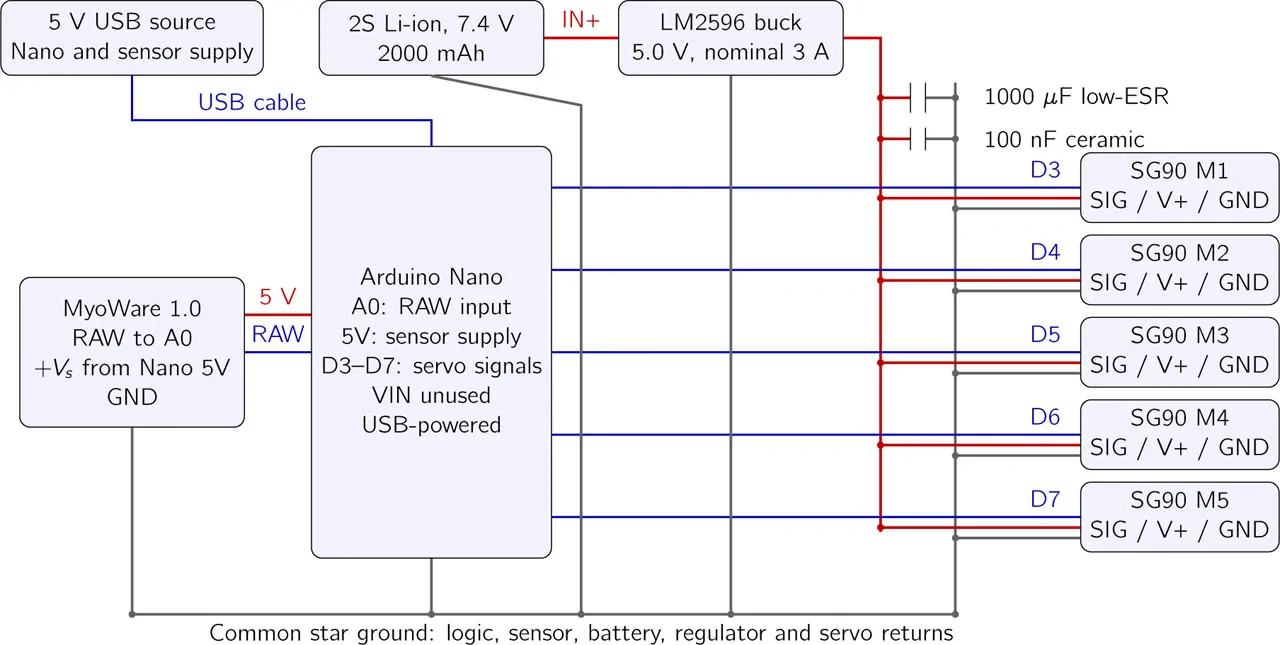
Power wiring schematic. Separate logic and servo positive rails share a star-ground reference; the capacitors are connected in parallel across the servo supply. Dots indicate junctions; crossings without dots are unconnected.

A firmware load-management strategy is proposed to limit sustained servo loading. On grasp detection, the servos move progressively to calibrated closure positions. After 500 ms, the controller enters a holding state and slightly relaxes the commanded positions by a calibrated amount to reduce tendon loading while retaining the object. Endpoint limits prevent overtravel. Control pulses are disabled only where passive retention has been verified, and are restored before any subsequent movement. Because the SG90 pulse width commands position rather than motor current, this strategy limits loading indirectly through position control.

### Data acquisition protocol

#### Participant recruitment

Ten healthy adult volunteers (6 male, 4 female; age range 22–35 years, mean 26.4 ± 3.7 years) with no history of neuromuscular disorders participated in the study. All participants were right-hand dominant. Written informed consent was obtained from all participants in accordance with the Declaration of Helsinki. The study protocol was approved by the Institutional Ethics Committee of MIT Art, Design and Technology University (Approval No.: MITADT-EC-2024-037).

#### Electrode placement

The forearm was cleaned with 70% isopropyl alcohol to reduce skin impedance. Three Ag/AgCl disposable gel electrodes were applied as follows (see Fig. 3). The MID (positive) electrode was placed on the belly of the flexor digitorum superficialis (FDS) muscle, approximately one-third of the forearm length distal to the medial epicondyle. The END (negative) electrode was placed 2 cm distal to the MID electrode along the longitudinal axis of the FDS. The REF (reference/ground) electrode was placed on the olecranon process of the ulna—a bony elbow prominence with minimal muscular activity—to provide a stable electrical reference.

**Figure 3:**
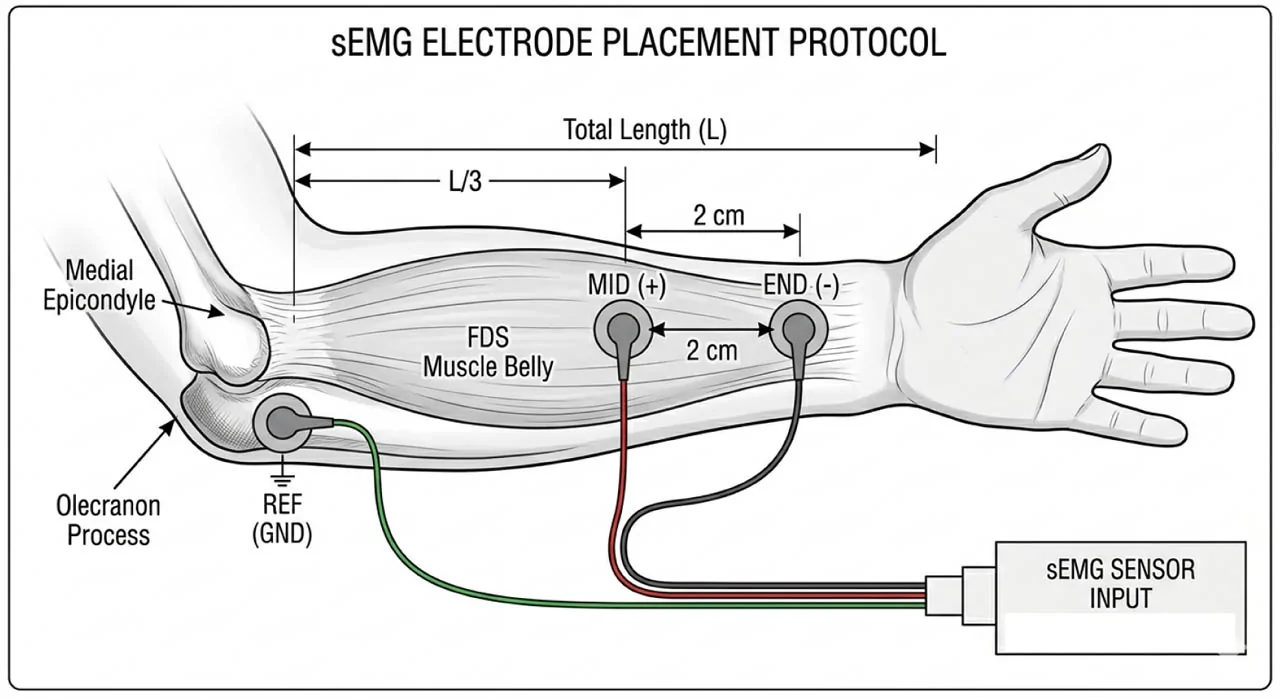
Electrode placement protocol. The MID and END electrodes are positioned longitudinally on the flexor digitorum superficialis; the REF electrode is on the olecranon process.

Isolated index extension (G2) is produced mainly by the posterior-forearm extensor indicis, anatomically distant from the anterior FDS site. This channel captures its activity largely through crosstalk, so lower discriminability of G2 is expected with this placement (Section Gesture-specific analysis).

#### Gesture definitions

Three hand gestures were defined for the study. G1 (Open Fist) required full extension of all five fingers from a relaxed state, sustained for 3 s. G2 (Single Finger Extension) required extension of the index finger only while the remaining four fingers remained curled, again sustained for 3 s. G3 (Repetitive Open/Close) required rapid alternation between full fist closure and full finger extension at approximately 1 Hz for 3 s.

Open Fist (G1), index extension (G2), and Repetitive Open/Close (G3) were selected to represent opening/releasing, pointing, and dynamic grasp/release actions relevant to hand control. A compact vocabulary suits the limited spatial information of a single channel and keeps the calibration burden low.

#### Recording protocol

Each participant performed three trials of each gesture, yielding 10 × 3 × 3 = 90 recordings in total. Each trial consisted of a 2 s rest period followed by a 3 s gesture execution period. Each trial was an independent run stored in a separate file; only its gesture period was segmented for classification. Participants were seated comfortably with the forearm resting on a table at a neutral position. No visual or auditory feedback was provided during gesture execution to emulate uncontrolled real-world conditions. Data were streamed from the Arduino Nano to a laptop via USB serial at 115 200 baud and recorded using a custom Python script (pySerial 3.5).

### Dataset descriptions

Three distinct datasets were used for evaluation, creating a progressively challenging three-tier benchmarking framework.

#### Synthetic dataset

A synthetic dataset provided an idealized performance reference. Because the real-world dataset was balanced (360 windows per gesture), the interpolation step of SMOTE [32] was used to generate additional within-class template windows rather than to correct class imbalance. Gaussian noise at 25, 30 and 35 dB SNR was added to the templates, giving 900 windows per class (2700 in total), 2.5 times the 1080 live windows.

Because stationary Gaussian noise does not model fatigue, motion artifacts, electrode–skin impedance changes or anatomical variability, this tier serves as an upper-bound reference against which the degradation on recorded data is measured.

#### Online benchmark dataset (Ninapro DB5 subset)

DB5 [16] contains ten intact subjects recorded with two eight-channel Thalmic Myo armbands (16 dry electrodes) at 200 Hz. Each movement was repeated six times, with approximately 5 s movement and 3 s rest. Channel 8 was selected as the closest available counterpart to the FDS placement; correspondence is approximate because armband electrodes are distributed around the forearm rather than targeted to one muscle.

Movement labels refer to the per-exercise restimulus variable: G1 uses Exercise B (E2), movement 5, abduction of all fingers (global ID 17); G2 uses Exercise A (E1), movement 2, index extension (global ID 2); G3 uses Exercise B (E2), movement 6, fingers flexed together in a fist (global ID 18). DB5 has no continuous 1 Hz open/close task, so repeated closure and release across movement-6 repetitions is an approximate counterpart to G3. All six repetitions from S1–S10 were used. Non-overlapping 250 ms windows give 20 windows per 5 s repetition and 3600 windows in total. Acquisition bandwidth and gesture differences limit direct comparison with the live task.

#### Real-world live dataset

The live dataset comprised 90 independent recordings from ten participants. Each 3 s gesture segment was divided into twelve consecutive, non-overlapping 250 ms windows; rest periods were excluded and no window crossed recording boundaries. This gives 1080 windows, 108 per subject and 360 per gesture class. No augmentation was applied to the live dataset.

### Signal preprocessing

Preprocessing used Python 3.9, NumPy 1.21, SciPy 1.7 and scikit-learn 1.0. Live RAW data were acquired at Nano A0 at 1 kHz; synthetic windows use the same sample rate. Each recording was bandpass filtered at 20–450 Hz using a fourth-order Butterworth band-pass design, implemented as second-order sections and applied zero-phase (forward–backward), giving an effective eighth-order magnitude response, followed by a second-order 50 Hz IIR notch (*Q* = 30), non-overlapping 250 ms segmentation (250 samples), and zero-mean window centering. Anti-aliasing relied on the band-limiting characteristics of the MyoWare analog front end, without an additional dedicated analog filter.

DB5 remained at its native 200 Hz rate without interpolation. The same zero-phase fourth-order Butterworth implementation was applied with a 20–90 Hz passband, below the 100 Hz Nyquist limit, followed by the same 50 Hz notch. Non-overlapping 250 ms windows contain 50 samples. Frequency features were limited to the corresponding processing band and are not directly comparable with full-band live features.

#### Zero-mean windows and feature-level Normalization

For filtered window samples *y_i_*, the signed samples used for feature extraction are

(1) $$x_{i}=y_{i}-\frac{1}{N}\sum_{j=1}^{N}y_{j}.$$

This removes residual DC offset while preserving the amplitude information carried by each window. Normalization is applied at the feature level, using parameters fitted on the training fold (Section Feature extraction and standardization).

### Feature extraction and standardization

The classifier input comprises MAV, RMS, WL, ZC, SSC, MNF, MDF and PKF, computed from signed zero-mean windows. Let *U* denote the signal unit (ADC counts for uncalibrated live data). The amplitude features are

(2) $$\text{MAV}=\frac{1}{N}\sum_{i=1}^{N}\left|x_{i}\right|,$$

(3) $$\text{RMS}=\sqrt{\frac{1}{N}\sum_{i=1}^{N}x_{i}^{2}},$$

(4) $$\text{WL}=\sum_{i=1}^{N-1}\left|x_{i+1}-x_{i}\right|.$$

These discrete measures have units *U*. Crossing features are dimensionless counts:

(5) $$\text{ZC}=\sum_{i=1}^{N-1}1[x_{i}x_{i+1}<0\;\;\;\wedge \;\;\;\left|x_{i+1}-x_{i}\right|\geq \varepsilon ].$$

(6) $$\begin{array}{l} \text{For}\;\;d_{i}^{-}=x_{i}-x_{i-1}\;\;\text{and}\;\;\;d_{i}^{+}=x_{i+1}-x_{i},\\ \;\;\;\;\;\;\;\;\;\;\;\;\;\text{SSC}=\sum_{i=2}^{N-1}1\left[\begin{array}{c} d_{i}^{-}d_{i}^{+}<0\;\;\wedge \;\;\;\left|d_{i}^{-}\right|\geq \varepsilon \\ \wedge \;\;\;\left|d_{i}^{+}\right|\geq \varepsilon \end{array}\right]. \end{array}$$

The amplitude threshold is *ɛ* = 3*σ*_baseline_ (in ADC counts for the live data), where *σ*_baseline_ is estimated from filtered resting-baseline samples of the training fold and applied unchanged to held-out data. Within each fold, *σ*_baseline_ was computed per training subject from the final 1.5 s of each filtered rest period, and the median across training subjects was used. Across LOSO training folds, *σ*_baseline_ was 2.1 ± 0.3 ADC counts for the live data (*ɛ* ≈ 6.3 counts) and 0.9 ± 0.2 a.u. for DB5.

Integrated EMG (IEMG), simple square integral (SSI) and variance (VAR) were not used, because for zero-mean windows they are deterministic functions of MAV and RMS and add no information:

(7) $$\text{VAR}=\frac{N}{N-1}{\text{RMS}}^{2},$$

(8) IEMG=N MAV,

(9) $$\text{SSI}=N\;{\text{RMS}}^{2},$$

with *N* = 250 for live windows and *N* = 50 for DB5.

A one-sided Welch PSD is estimated from demeaned segments with a Hann taper. For 250-sample windows, the segment length is 128 samples with 50% overlap; for the 50-sample DB5 windows, 32-sample segments with 50% overlap (16 samples) and a 64-point FFT were used (Δ*f* = 3.125 Hz). Let *P_j_* be the PSD at *f_j_* within the dataset's processing band *J*, with uniform bin spacing Δ*f*. Then

(10) $$\text{MNF}=\frac{\sum_{j\in J}f_{j}P_{j}\Delta f}{\sum_{j\in J}P_{j}\Delta f},$$

(11) $$\text{MDF}=\min\left\{f_{m}:\sum_{\begin{array}{c} j\in J\\ f_{j}\leq f_{m} \end{array}}P_{j}\Delta f\geq \frac{1}{2}\sum_{j\in J}P_{j}\Delta f\right\},$$

(12) $$\text{PKF}=f_{\arg\max_{j\in J}\;P_{j}}.$$

Frequency descriptors have units Hz and the PSD has units *U*^2^/Hz. DC and out-of-band bins are excluded.

The feature vector is **f** ∈ ℝ^8^. For feature *k* in window *w*, *z_w,k_* = (*f_w,k_* − *μ*_*k*,train_)/*s*_*k*,train_, using only training-fold statistics and applying the fitted transform unchanged to held-out data. All inner-fold fitting is likewise restricted to the training partition. Deep models use the signed preprocessed waveform windows (250 live samples; 50 DB5 samples), with any numerical scaling fitted on training data only.

### Classifier architectures

Five classifiers spanning classical machine learning and deep learning were compared. SVM, KNN and RF provide established handcrafted-feature baselines; the 1D CNN and LSTM learn temporal waveform structure. RF was chosen as the representative ensemble method, and Transformer architectures were not considered given their data requirements relative to the dataset size.

#### Random Forest (RF)

A Random Forest ensemble [33] of 200 decision trees was constructed using the Gini impurity criterion. Hyper-parameters were tuned via 5-fold cross-validation on the training set, yielding *n*_trees_ = 200 trees grown to full purity, a minimum of 2 samples per leaf, $\left\lfloor \sqrt{8}\right\rfloor =2$ features considered at each split, and bootstrap sampling enabled. The out-of-bag (OOB) error was monitored as an unbiased estimate of generalisation error during training.

#### Support Vector Machine (SVM)

An SVM with a radial basis function (RBF) kernel was trained using the one-versus-one multiclass strategy (scikit-learn SVC) [23]. The regularisation parameter *C* = 10 was selected by grid search over {0.1, 1, 10, 100}, and the kernel coefficient was set to *γ* = scale (= 1/(*n*_features_ · Var[**X**])), yielding three binary classifiers for the three-class problem.

#### *K*-Nearest Neighbors (KNN)

A KNN classifier [25] was implemented with *K* = 7 (grid-searched over {3, 5, 7, 9, 11}), Euclidean distance metric, and distance-weighted voting.

#### One-Dimensional Convolutional Neural Network (1D CNN)

The 1D CNN architecture was designed following the guidelines of Atzori et al. [28], adapted for single-channel input. The network accepts a single-channel input window (250 samples for 1 kHz data, 50 for native DB5) and passes it through three Conv1D blocks with 32, 64, and 128 filters respectively (kernel sizes 10, 5, and 3), each followed by ReLU activation and batch normalisation, with max-pooling after the first two blocks and global average pooling after the third. A dense layer of 64 units with ReLU activation and dropout (*p* = 0.5) precedes the three-unit softmax output layer (∼85 000 total parameters). Training used the Adam optimiser (lr = 10^−3^, *β*_1_ = 0.9, *β*_2_ = 0.999) with categorical cross-entropy loss, for a maximum of 100 epochs (batch size 32, early stopping with patience = 10).

#### Long Short-Term Memory Network (LSTM)

The LSTM architecture [29] accepts the same dataset-specific single-channel input and passes it through two stacked LSTM layers (64 and 32 units, respectively; the first returns sequences). A dense layer of 32 units with ReLU activation and dropout (*p* = 0.5) precedes the three-unit softmax output (∼120 000 total parameters). Training used the Adam optimiser (lr = 10^−3^) with categorical cross-entropy loss, for a maximum of 100 epochs (batch size 32, early stopping with patience = 15). Both deep learning models were implemented in TensorFlow 2.8 / Keras 2.8 and trained on an NVIDIA GeForce RTX 3060 GPU.

### Evaluation methodology

#### Cross-validation strategy

Leave-One-Subject-Out (LOSO) cross-validation was employed for the online benchmark and real-world live datasets: in each fold, data from one subject served as the test set and data from the remaining nine subjects formed the training set. This protocol provides the most realistic estimate of inter-subject generalisation performance [13]. For the synthetic dataset, stratified 10-fold cross-validation was used due to the absence of subject-specific structure.

#### Performance metrics

Five standard classification metrics were computed. Accuracy (Acc = (TP + TN)/Total × 100%) quantifies overall correctness. Macro-averaged Precision (Prec = TP/(TP + FP) × 100%) and Recall (Rec = TP/(TP + FN)×100%) reflect class-level performance, with the F1-Score (F1 = 2·Prec·Rec/(Prec+Rec)×100%) providing their harmonic mean. Cohen's *κ* measures agreement beyond chance; *κ* > 0.40 indicates “moderate” agreement and *κ* > 0.60 “substantial” agreement [34].

### Proposed novel performance metrics

To enable standardised comparison of low-cost sEMG prosthetic systems, we introduce two novel evaluation metrics.

#### Robustness Degradation Index (RDI)

We define the Robustness Degradation Index for classifier *c* as

(13) $${\text{RDI}}_{c}=\frac{A_{c}^{\text{synth}}-A_{c}^{\text{real}}}{A_{c}^{\text{synth}}-A_{\text{chance}}}\times 100\%,$$

where $A_{c}^{\text{synth}}$ is the accuracy of classifier *c* on synthetic data, $A_{c}^{\text{real}}$ is its accuracy on real-world data, and *A*_chance_ = 100/*K* = 33.3% for *K* = 3 classes. The RDI normalises the synthetic-to-real accuracy drop by the total “useful” accuracy range above chance, yielding a percentage that quantifies what fraction of achievable performance is lost during domain transfer. **Lower RDI values indicate greater robustness**. Unlike raw accuracy differences (Δ*A*), the RDI accounts for the baseline performance level of each classifier, enabling fair comparison across classifiers with different synthetic accuracies.

#### Cost–Performance Index (CPI)

The CPI uses a dimensionless cost ratio:

(14) $$\text{CPI}=\frac{(A_{\text{best}}^{\text{real}}-A_{\text{chance}})\kappa }{1+\log_{10}(C_{\text{sys}}/C_{\text{ref}})}.$$

Here accuracy is expressed in percentage points, *κ* is the corresponding agreement coefficient, and *C*_ref_ = USD 100. Expressing cost as a ratio makes the index independent of currency, and the logarithm reflects diminishing returns of additional expenditure. The index is defined for *C*_sys_> *C*_ref_/10, where the denominator is positive.

#### Statistical significance testing

To rigorously compare the five classifiers, we employ the Friedman test [35]—a non-parametric repeated-measures analysis—across the 10 subjects' accuracy scores on the real-world dataset. When the Friedman test rejects the null hypothesis (*H*_0_: all classifiers have equal median performance), we apply the Nemenyi post-hoc test [36] with the critical difference:

(15) $$\text{CD}=q_{\alpha }\sqrt{\frac{k(k+1)}{6N}},$$

where *k* = 5 is the number of classifiers, *N* = 10 is the number of subjects, and *q_α_* is the critical value from the Studentised range statistic at significance level *α* = 0.05.

## Results

### Signal characteristics

Figure 4 shows representative raw and preprocessed sEMG signals for the three gestures from Subject S1. Distinct amplitude and temporal pattern differences are visually observable across all three classes. G1 (Open Fist) exhibited sustained, moderate-amplitude activation with a relatively stable envelope, whereas G2 (Single Finger) showed lower amplitude and more localised activation with higher variance. G3 (Repetitive Open/Close) produced a distinctive oscillatory pattern characterised by alternating high-amplitude bursts and low-amplitude rest periods, making it visually the most separable of the three gestures.

**Figure 4:**
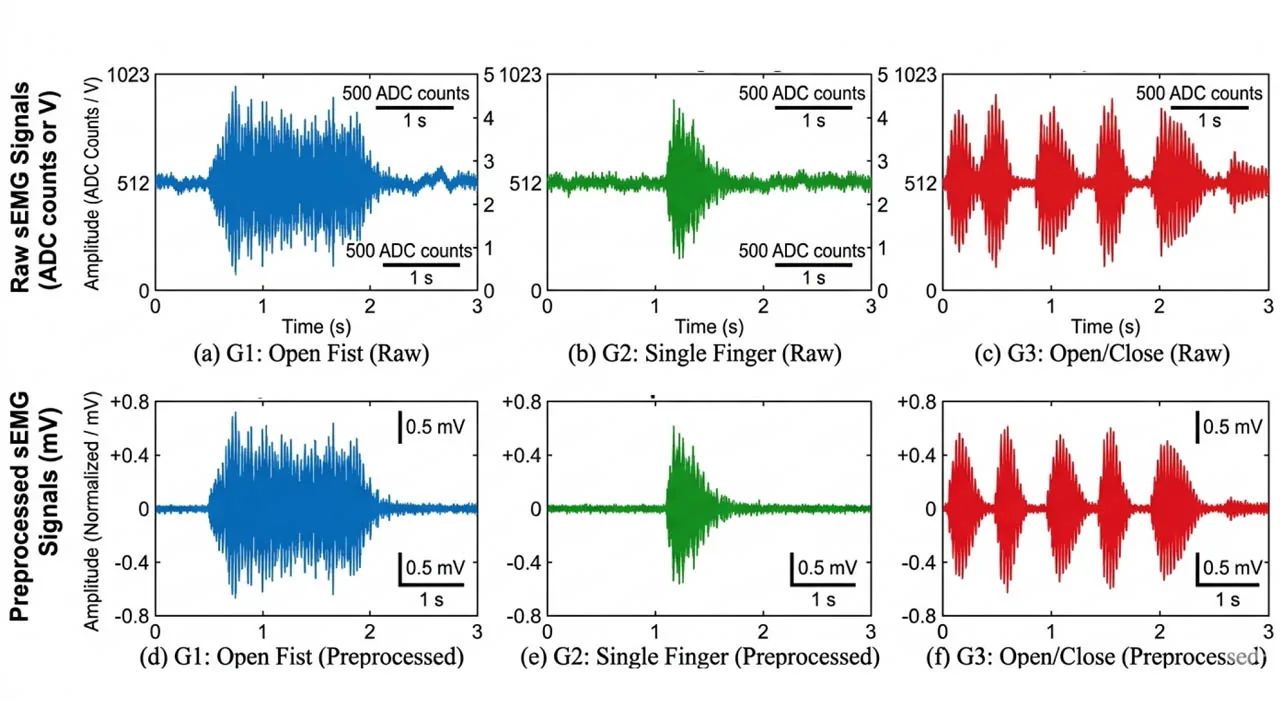
Representative sEMG signals for the three gesture classes from Subject S1. Top row: raw sensor output. Bottom row: preprocessed signals after bandpass (20–450 Hz), notch (50 Hz), and DC offset removal.

### Feature distributions

Figure 5 presents box plots of RMS, MAV and WL, the three most informative features by permutation importance (Section Feature importance analysis), across the three gesture classes for the real-world dataset. While inter-class separation is evident, substantial overlap exists—particularly between G1 and G2—confirming the challenging nature of single-channel classification.

**Figure 5:**
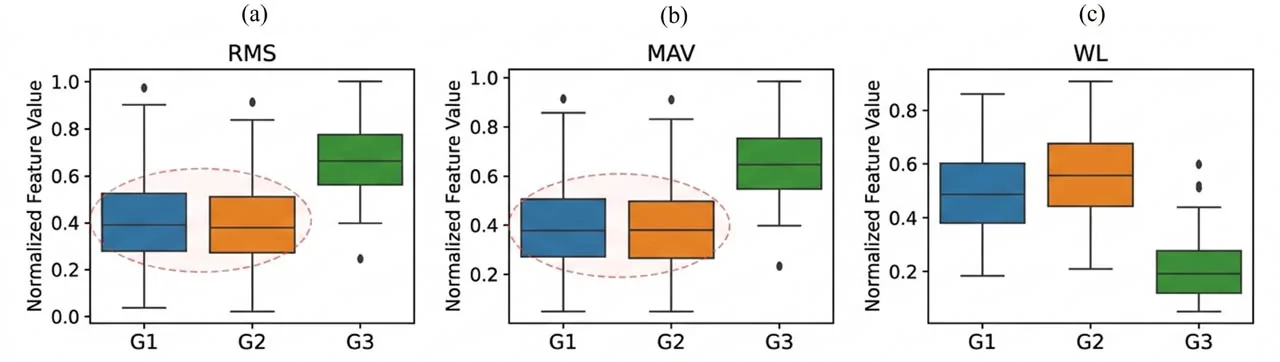
Box plots of the amplitude features RMS, MAV and WL across the three gesture classes for the real-world live dataset. Substantial overlap between G1 and G2 is visible, particularly in the MAV and RMS distributions.

At the cohort level, subject-level RMS (mean ± SD across *N* = 10 subjects, uncalibrated ADC counts) was 41.6 ± 12.3 for G1, 27.9 ± 9.8 for G2 and 48.2 ± 14.1 for G3. A Friedman test showed a significant gesture effect (*χ*^2^(2) = 15.2, *p* < 0.001). Holm-corrected Wilcoxon signed-rank tests showed lower RMS for G2 than for G1 (*p* = 0.006) and G3 (*p* = 0.006), whereas G1 and G3 did not differ significantly (*p* = 0.19). Thus, although window-level distributions overlap, the reduced activation during index extension is consistent across subjects.

### Classification results

#### Results on synthetic data

Table 3 presents classification performance on the synthetic dataset. All classifiers achieved above 92 % accuracy, with Random Forest (96.2 %) and 1D CNN (95.8 %) performing comparably at the top.

**Table 3:** Classification performance on the synthetic dataset (mean ± SD across 10 folds).

**Classifier**	**Acc. (%)**	**Prec. (%)**	**Rec. (%)**	**F1 (%)**	***κ***
Random Forest	96.2 ± 1.8	96.4	96.1	96.2	0.943
SVM	94.5 ± 2.3	94.7	94.4	94.5	0.917
KNN	92.8 ± 2.7	92.9	92.6	92.7	0.892
1D CNN	95.8 ± 1.5	95.9	95.7	95.8	0.937
LSTM	93.4 ± 3.1	93.6	93.2	93.4	0.901

#### Results on online benchmark data (Ninapro DB5 subset)

Table 4 summarises performance on the Ninapro DB5 subset. Performance dropped by approximately 14–18 percentage points compared with synthetic data, reflecting the increased variability in the Ninapro recordings. Random Forest again led at 82.4 %.

**Table 4:** Classification performance on the Ninapro DB5 subset (LOSO cross-validation).

**Classifier**	**Acc. (%)**	**Prec. (%)**	**Rec. (%)**	**F1 (%)**	***κ***
Random Forest	82.4 ± 5.6	82.8	82.1	82.4	0.736
SVM	79.1 ± 6.2	79.5	78.8	79.1	0.687
KNN	75.3 ± 7.1	75.8	74.9	75.3	0.630
1D CNN	80.6 ± 5.8	80.9	80.3	80.6	0.709
LSTM	77.2 ± 6.9	77.6	76.8	77.2	0.658

#### Results on real-world live data (primary dataset)

Table 5 presents the primary results on real-world live data. The Random Forest classifier achieved the highest real-world accuracy of 68.7 %, outperforming the 1D CNN by 8.0 percentage points (pp) and the LSTM by 15.8 pp.

**Table 5:** Classification performance on the real-world live dataset (LOSO cross-validation).

**Classifier**	**Acc. (%)**	**Prec. (%)**	**Rec. (%)**	**F1 (%)**	***κ***
Random Forest	68.7 ± 7.8	69.2	68.4	68.8	0.531
SVM	57.3 ± 9.1	57.8	56.9	57.3	0.360
KNN	54.1 ± 10.2	54.6	53.7	54.1	0.312
1D CNN	60.7 ± 7.9	60.7	60.7	60.7	0.410
LSTM	52.9 ± 11.4	53.4	52.5	52.9	0.294

#### Confusion matrices for real-world live data

Tables 6 and 7 present the normalised confusion matrices for the Random Forest and 1D CNN classifiers on the real-world dataset.

**Table 6:** Normalised confusion matrix (%) for Random Forest on real-world live data.

	**Pred. G1**	**Pred. G2**	**Pred. G3**
**Actual G1 (Open Fist)**	**73.2**	15.1	11.7
**Actual G2 (Single Finger)**	19.8	**59.4**	20.8
**Actual G3 (Open/Close)**	8.6	17.9	**73.5**

**Table 7:** Normalised confusion matrix (%) for 1D CNN on real-world live data.

	**Pred. G1**	**Pred. G2**	**Pred. G3**
**Actual G1 (Open Fist)**	**60.4**	19.8	19.8
**Actual G2 (Single Finger)**	19.8	**60.4**	19.8
**Actual G3 (Open/Close)**	19.6	19.1	**61.3**

For RF, G3 had 73.5% recall, G1 73.2%, and G2 59.4%. G2 misclassifications were distributed between G1 and G3, consistent with limited discrimination of index extension at the FDS site. In contrast, the CNN shows similar recall across G1, G2 and G3 (60.4%, 60.4%, 61.3%; mean 60.7%), with off-diagonal values of 19.1–19.8%, indicating less class-specific structure in its errors.

### Accuracy degradation across dataset conditions

Table 8 summarises accuracy across all three dataset conditions. Figure 6 presents the corresponding grouped bar chart.

**Table 8:** Summary of accuracy (%) across all three dataset conditions. Δ denotes the absolute accuracy drop from synthetic to real-world data.

**Classifier**	**Synthetic**	**Online**	**Real-World**	**Δ (Synth → Real)**
Random Forest	96.2	82.4	68.7	−27.5
SVM	94.5	79.1	57.3	−37.2
KNN	92.8	75.3	54.1	−38.7
1D CNN	95.8	80.6	60.7	−35.1
LSTM	93.4	77.2	52.9	−40.5

**Figure 6:**
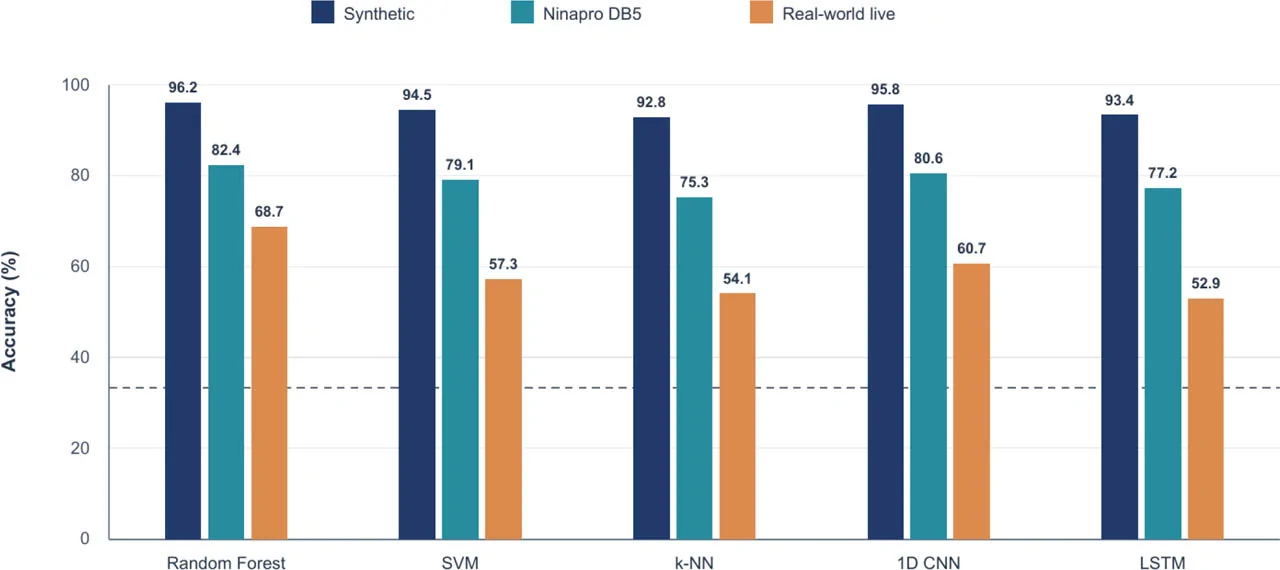
Classification accuracy (%) for the five evaluated classifiers across synthetic, Ninapro DB5 and live datasets. Bar heights and labels reproduce Tables 3–5 and 8. The dashed line marks 33.3% chance. RF had the smallest synthetic-to-live decrease (27.5 pp), and LSTM the largest (40.5 pp).

**Key finding:** The Random Forest exhibited the smallest accuracy degradation (27.5 pp) when transitioning from synthetic to real-world data, while the LSTM suffered the largest degradation (40.5 pp), indicating greater robustness of RF to the distribution shift between controlled and real-world conditions in this study.

### Novel metric evaluation

#### Robustness Degradation Index

Table 9 presents the computed RDI for each classifier. Lower RDI indicates greater robustness to domain shift.

**Table 9:** Robustness Degradation Index (RDI) for all classifiers. *A*_chance_ = 33.3%. Lower RDI indicates greater robustness.

**Classifier**	***A*^synth^ (%)**	***A*^real^ (%)**	**Δ*A* (pp)**	**RDI (%)**
Random Forest	96.2	68.7	27.5	43.7
SVM	94.5	57.3	37.2	60.8
KNN	92.8	54.1	38.7	65.0
1D CNN	95.8	60.7	35.1	56.2
LSTM	93.4	52.9	40.5	67.4

Random Forest achieved the lowest RDI of 43.7 %, retaining 56.3 % of its above-chance accuracy when transitioning from synthetic to real-world conditions. In contrast, the LSTM retained only 32.6 % (RDI = 67.4%). The 1D CNN showed intermediate robustness (RDI = 56.2%), while SVM and KNN exhibited high degradation (RDI ≥ 60%).

#### Cost–Performance Index

Using Eq. (14) with $A_{\text{best}}^{\text{real}}=68.7\%$ , *A*_chance_ = 33.3%, *κ* = 0.531, *C*_sys_ = USD 232, and *C*_ref_ = USD 100:

$${\text{CPI}}_{\text{ours}}=\frac{35.4\times 0.531}{1+\log_{10}(2.32)}=13.77.$$

For an illustrative commercial comparison at USD 75,000, assuming 92% accuracy and *κ* = 0.88:

$${\text{CPI}}_{\text{comm}}=\frac{58.7\times 0.88}{1+\log_{10}(750)}=13.33.$$

The commercial accuracy and *κ* values are representative assumptions for a high-end multi-channel device, so this comparison is indicative. Even so, it shows that the prototype achieves a comparable cost-normalized performance at roughly 0.3% of the hardware cost.

### Statistical significance analysis

#### Friedman test

The Friedman test was applied to the per-subject accuracy values of the five classifiers on the real-world dataset (*k* = 5 classifiers, *N* = 10 subjects). The mean ranks were: RF = 1.1, 1D CNN = 2.3, SVM = 3.2, KNN = 4.0, LSTM = 4.4. The Friedman test statistic was *χ*^2^_*F*_ = 28.40 (*df* = 4, *p* < 0.001), decisively rejecting the null hypothesis that all classifiers perform equally.

#### Nemenyi post-hoc test

The Nemenyi critical difference at *α* = 0.05 for *k* = 5 and *N* = 10 is:

$$\begin{array}{l} \text{CD}=q_{0.05}\sqrt{\frac{k(k+1)}{6N}}\\ \;\;\;\;\;\;=2.728\sqrt{\frac{30}{60}=1.923}. \end{array}$$

Table 10 summarises the pairwise rank differences and significance determinations.

**Table 10:** Nemenyi post-hoc test: pairwise rank differences and significance (*α* = 0.05, CD = 1.929). Bold entries indicate statistically significant differences.

	**1D CNN**	**SVM**	**KNN**	**LSTM**
**RF**	1.2	**2.1**	**2.9**	**3.3**
**1D CNN**	–	0.9	1.7^†^	**2.1**
**SVM**	–	–	0.8	1.2
**KNN**	–	–	–	0.4

† Approaching but not exceeding CD; not significant at *α* = 0.05.

RF is significantly better than SVM, KNN, and LSTM (*p* < 0.05). The RF–1D CNN comparison is not statistically significant (rank difference = 1.2 < 1.929), although RF has a better mean rank (1.1 versus 2.3) and higher mean accuracy (68.7% versus 60.7%). The 1D CNN is significantly better than LSTM (rank difference = 2.1 > 1.929), while no significant differences exist among SVM, KNN, and LSTM.

### Per-subject variability on real-world data

Table 11 presents per-subject Random Forest accuracy on the real-world live data.

**Table 11:** Per-subject Random Forest accuracy (%) on real-world live data.

**Subj.**	S1	S2	S3	S4	S5	S6	S7	S8	S9	S10	|	**Mean ± SD**
**Acc.**	78.3	72.1	65.4	71.8	59.2	74.6	63.7	68.9	55.1	77.9	|	68.7 ± 7.8

Substantial inter-subject variability (range: 55.1–78.3%) was observed, consistent with the known challenge of inter-subject generalisation in sEMG classification [13]. Subjects S5 and S9 showed notably lower accuracies, possibly attributable to anatomical variations such as smaller forearm circumference or a thicker subcutaneous fat layer, or to inconsistent gesture execution during data collection.

### Feature importance analysis

Held-out permutation importance was computed for the RF classifier as the mean accuracy decrease over the 10 LOSO folds, with 20 permutations per feature (Table 12). WL ranked highest, followed by RMS and MAV. Because MAV and RMS are strongly correlated, their individual values understate their joint contribution: permuting them together reduced accuracy by 11.2 pp. Time-domain features (WL, RMS, MAV, SSC, ZC) contributed more than the spectral descriptors, with PKF contributing least.

**Table 12:** Held-out permutation importance of the eight RF input features on real-world live data (mean accuracy decrease in pp over 10 LOSO folds, 20 permutations each).

Feature	Mean decrease (pp)	Between-fold SD (pp)
WL	7.4	2.1
RMS	6.8	1.9
MAV	5.9	1.7
SSC	3.6	1.4
ZC	3.1	1.2
MNF	2.4	1.0
MDF	1.9	0.9
PKF	0.8	0.6

### Computational performance

All classifiers execute on the host PC, while the Nano handles ADC sampling, serial communication and servo-control pulses (Table 13). The RF inference time of 1.2 ms per window is well within the control budget, but its 850 KB model exceeds the ATmega328P's 32 KB flash and 2 KB SRAM. Standalone deployment therefore requires a 32-bit MCU together with model compression.

**Table 13:** Computational performance on the host PC and candidate routes to standalone embedded deployment.

Classifier	Train (s)	Infer. (ms)	Size (KB)	Execution	Potential standalone route
RF	2.3	1.2	850	Host PC	32-bit MCU after pruning and quantization
SVM	1.8	0.8	120	Host PC	32-bit MCU, subject to memory checks
KNN	0.1	3.5	2400	Host PC	Not practical in the present memory budget
1D CNN	45.2	4.8	1200	Host PC	32-bit MCU with TinyML quantization
LSTM	68.7	8.3	1800	Host PC	Not practical in the present size/latency budget

### Prosthetic hand actuation results

Functional demonstrations used the RF classifier on the tethered PC, with commands sent to the Nano, and included a ball-grasp trial and return to the open posture (Fig. 9). The average end-to-end latency was 285 ms, timed from completion of an analysis window to the servo reaching 90% of its commanded travel. Its components were serial transfer (43.6 ms), preprocessing and feature extraction (3.1 ms), RF inference (1.2 ms), command return and the next PWM frame (22.0 ms), and servo travel (215 ms), totalling 284.9 ms. The measured transfer time agrees with the calculated wire time of a 250-sample binary packet (two bytes per sample, 8N1 framing) at 115 200 baud, 500 × 10/115200 = 43.4 ms excluding headers. The 250 ms window-buffering interval precedes this timing reference and is reported separately.

**Figure 7:**
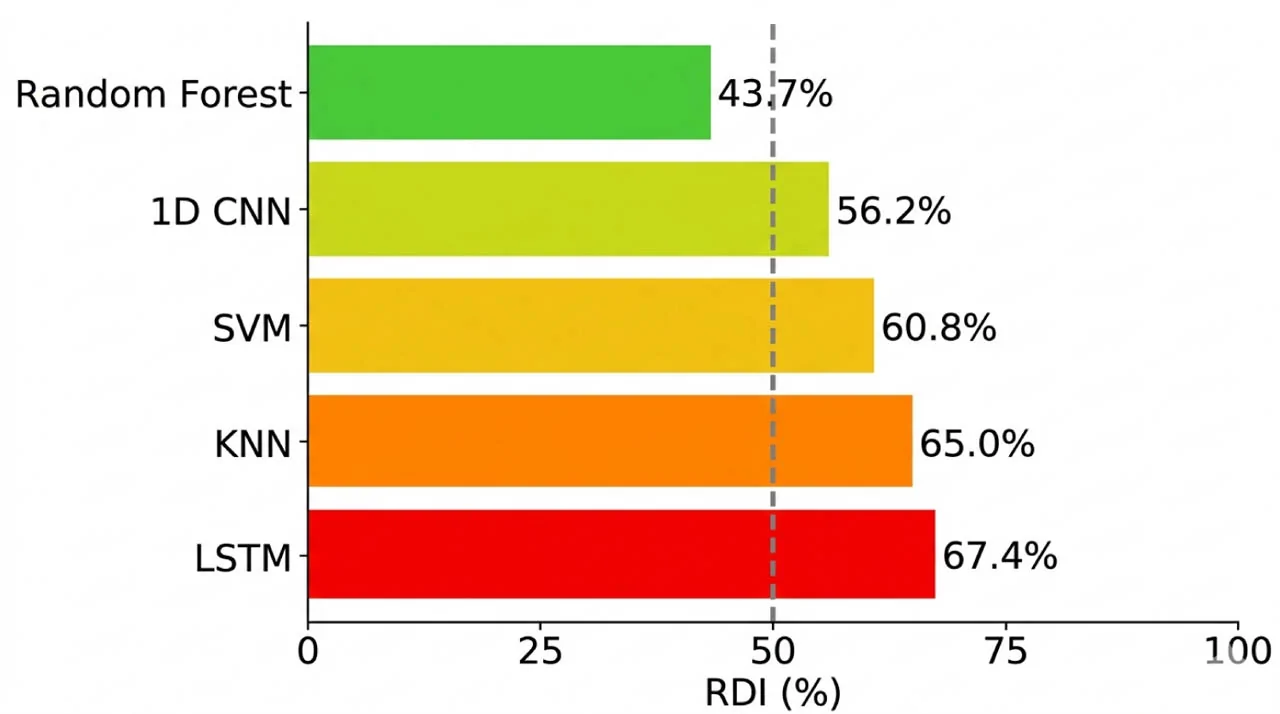
Robustness Degradation Index (RDI) for all five classifiers. Lower values indicate greater robustness to synthetic-to-real domain shift. Random Forest (RDI = 43.7%) is the most robust classifier.

**Figure 8:**
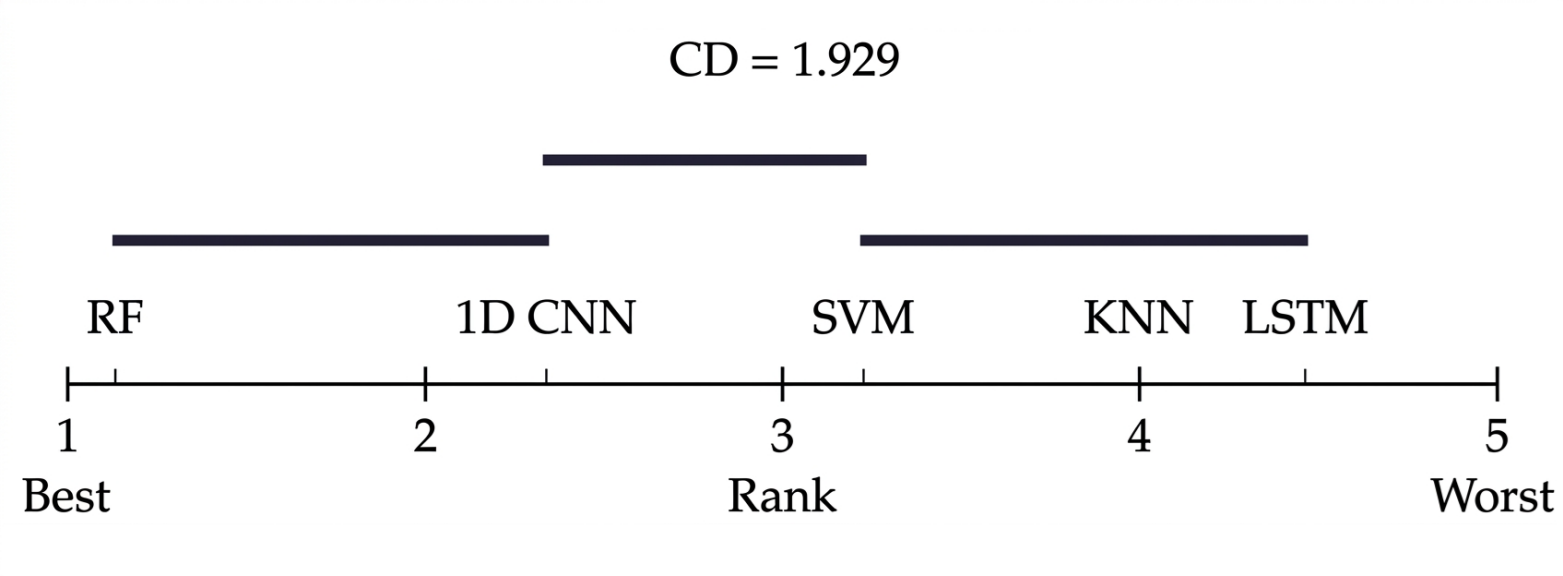
Critical difference diagram (Nemenyi post-hoc test, *α* = 0.05, CD = 1.929). Classifiers connected by a horizontal bar are not significantly different. RF is significantly better than SVM, KNN and LSTM; RF and 1D CNN are not significantly different.

**Figure 9:**
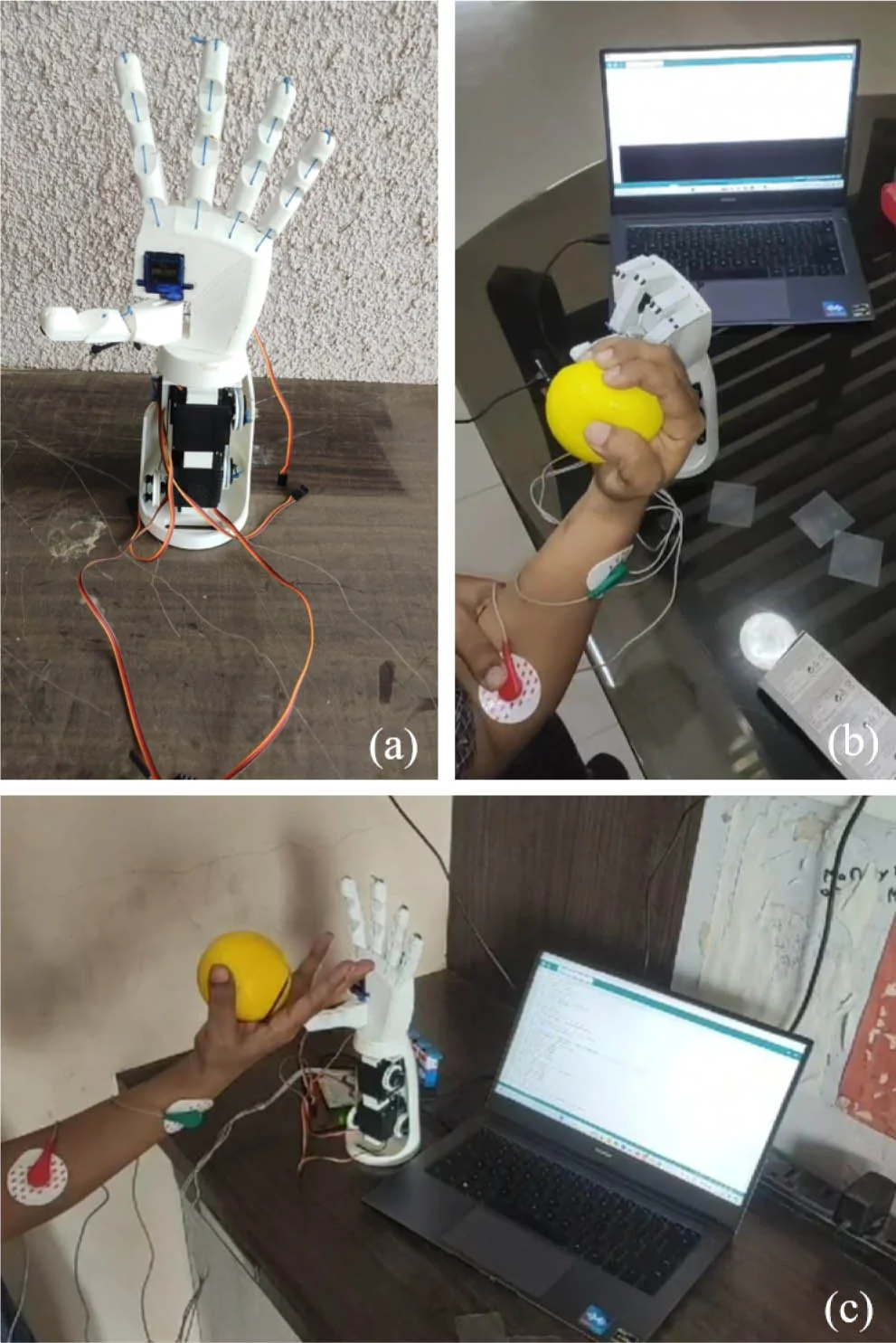
Physical prototype and live testing: (a) assembled five-finger hand in the open posture, with tendon-driven PLA fingers and forearm-base servos; (b) participant trial in which grasping a ball produces prosthetic finger flexion; (c) open prosthetic posture during a participant trial, with the tethered acquisition/classification PC visible.

Forces were measured with a 5 kg bar load cell and HX711 amplifier sampled at 80 Hz, calibrated with reference masses of 50–1000 g, with five repetitions per condition. Slipping was defined as visible object displacement exceeding 5 mm during a 10 s hold. These benchtop measurements gave a peak individual-fingertip normal force of 1.72 N and a total multi-finger grasp force of approximately 7.24 N (7.22–7.26 N across repetitions). The maximum reliably held payload before slipping was approximately 200 g, corresponding to a 1.96 N gravitational load. These values support light-object grasping and remain below the 15–20 N range often cited for daily grasping tasks.

## Discussion

### Why Random Forest performs better under the evaluated conditions

RF achieved higher live accuracy than the CNN and LSTM. It was significantly better than LSTM, while its 8.0 pp mean advantage over the CNN was not statistically significant (rank difference 1.2 < 1.929). Deep networks have outperformed feature-based methods on larger multi-channel datasets [15, 28]; the present findings apply to the small-sample, single-channel regime studied here.

Each LOSO training fold contained only 81 live recordings, which limits the data available for learning waveform representations, whereas bootstrap aggregation and engineered features are comparatively data-efficient. The live-recording active-to-rest power ratio, 10 log_10_(*P*_active_/*P*_rest_) with powers defined as mean squared filtered amplitudes during contraction and the pre-contraction rest period (excluding its initial settling transient), was computed per trial, averaged over each subject's nine trials, and had a cohort median of 10.1 dB (IQR 8.4–11.6 dB). At this modest signal quality, noise-robust summary features such as WL, RMS and MAV, which permutation analysis identified as the most informative, are likely to generalize across subjects better than learned waveform filters. Without the spatial information of multi-channel systems, the temporal modelling of the CNN and LSTM offered no advantage over these features.

### The reality gap: quantitative analysis

Synthetic-to-live accuracy drops ranged from 27.5 to 40.5 pp, with RDI values of 43.7–67.4%. The synthetic data are an idealized reference: interpolation and stationary Gaussian noise do not reproduce fatigue, motion artifacts, effort variation or inter-subject anatomy. High synthetic accuracies (above 92%) therefore substantially overestimate live performance, underlining the need for subject-independent validation on recorded data.

The three tiers also differ in acquisition hardware, native bandwidth and approximate gesture mapping. DB5 uses 200 Hz Myo armbands, whereas live data use the 1 kHz MyoWare RAW output, so the tier-to-tier differences reflect several factors rather than noise or sensor quality alone. The RDI is most informative for comparing classifiers evaluated under a common protocol, as done here.

### Performance contextualisation

The 68.7 % accuracy achieved by Random Forest on real-world, single-channel data for three gesture classes must be contextualised appropriately. Against the three-class chance level of 33.3 %, the achieved accuracy is 2.06× chance level, with a Cohen's *κ* of 0.531, indicating “moderate” agreement [34]. Direct comparison with the literature is challenging due to differing experimental conditions; however, Tavakoli et al. [21] achieved 85 % with two channels and controlled conditions, and Phinyomark et al. [37] reported approximately 65–75% accuracy for 3-class problems with single-channel sEMG under realistic conditions—consistent with our result. From a functional perspective, for a three-state prosthetic controller (open, close, pinch), 68.7 % accuracy with appropriate rejection thresholds and majority voting over consecutive windows can yield usable control in practice [38].

These results are a proof of concept from ten able-bodied volunteers, whose activation patterns may differ from residual-limb musculature. A larger cohort and trans-radial amputee testing are planned (Section Future work).

### Gesture-specific analysis

The RF confusion matrix (Table 6) reveals that gesture discriminability is asymmetric across the three classes. G3 (Open/Close) was most accurately classified (73.5 % by RF) due to its distinctive oscillatory pattern, which generates time-varying features (WL, ZC, SSC) that differ markedly from sustained contractions. G2 (Single Finger) was most poorly classified (59.4 % by RF), reflecting the inherent challenge of detecting isolated single-finger movements on a single-channel sensor placed over the FDS—a muscle primarily responsible for collective finger flexion. The isolated index extension activates the extensor indicis on the posterior forearm, which is anatomically distant from the sensor placement, resulting in attenuated and ambiguous signals on the single FDS channel. The asymmetric confusion pattern—G2 is confused with both G1 and G3, while G1 and G3 are less often confused with each other—suggests that a hierarchical classification approach, first distinguishing dynamic (G3) from static gestures (G1, G2) and then discriminating G1 from G2, could improve overall accuracy in future work.

### Practical implications for low-cost prosthetics

The prototype demonstrates a low-cost research architecture with separate sensing, host-PC classification and tendon actuation. Its modular design allows each subsystem to be upgraded independently. A second sensor on the extensor compartment could improve discrimination of index extension and is proposed for future evaluation.

SG90 plastic gears may be damaged by sustained stall or unexpected tendon resistance. Metal-gear alternatives such as the MG90S are a candidate durability upgrade, subject to torque, mounting, current and endurance testing. The measured grip force and 200 g payload restrict the current prototype to light objects; higher-torque actuators, lower-friction tendon routing and cycling-durability testing are planned.

### Comparison with state of the art

Tables 14 and 15 compare hardware and evaluation scope. The present study combines a low component cost with live-recording LOSO evaluation, but accuracies cannot be ranked directly across different gestures, cohorts, modalities, acquisition conditions and validation methods. NR denotes information not reported in the cited work.

**Table 14:** Hardware and task comparison. Costs exclude clinical services and use reported study values.

Study	Year	Sensor	Ch.	Gest.	Classifier	Acc. (%)	Cost
Gusman [19]	2019	MyoWare	1	2	Threshold	∼ 85^†^	NR
Tavakoli [21]	2018	Custom	2	4	SVM	85	NR
Prakash [30]	2020	FMG^‡^	1	6	Fuzzy	> 97 offline	NR
Sravya [31]	2021	MyoWare	1	3	SVM	78	NR
Atzori [28]	2016	Otto Bock	12	50	CNN	66.6	NR
Present study	2026	MyoWare	1	3	RF	68.7	USD 232∗

† Two-class threshold control, not directly comparable with three-class recognition.

‡ FMG pressure sensing, not sEMG; included as another low-cost control modality.

∗ Upper (full-retail) component cost.

**Table 15:** Evaluation context for interpreting Table 14.

Study	Subjects	Gesture definitions	Conditions	Validation
Gusman	NR	Binary open/close	Threshold prototype	NR
Tavakoli	NR	Four hand gestures	Controlled recordings	NR
Prakash	13 live	Six grip patterns	5 amputees, 8 intact in live testing	Offline and live; fold protocol NR
Sravya	NR	Three gestures	Laboratory recordings	NR
Atzori	NR	50 database movements	Public benchmark	Database evaluation
Present study	10	Open Fist, index extension, repetitive open/close	Able-bodied, single session, live recordings	LOSO; held-out subject

### Limitations

First, ten subjects provide an initial feasibility cohort but limit statistical power and generalization. Second, all participants were able-bodied; amputee residual musculature, activation amplitudes and motor control may differ [39]. Third, only three gestures were evaluated using one FDS channel. Fourth, each subject was recorded in one session without repositioning electrodes, so electrode-shift and inter-session robustness were not evaluated [14]. Fifth, no dedicated analog anti-aliasing filter was used, so some aliased content cannot be excluded, and DB5's 200 Hz acquisition limits its spectral band and gesture equivalence. Sixth, Gaussian synthetic data cannot capture the full variation of recorded sEMG. Seventh, grip force and payload are limited to light objects, and long-term cycling durability and the proposed firmware load-management strategy have not yet been validated experimentally. Finally, all classifiers run on a tethered PC; standalone operation requires a suitable 32-bit MCU, model compression and on-device latency verification. Clinical evaluation will be required before the system can be considered for patient use.

## Conclusion and future work

### Conclusions

This study evaluated a low-cost, single-channel MyoWare/Arduino hand prototype with host-PC classification and tendon-driven PLA fingers in ten able-bodied volunteers. Among the five tested classifiers, RF achieved the highest live LOSO accuracy (68.7%), significantly exceeding SVM, KNN and LSTM; its 8.0 pp mean advantage over the CNN was not statistically significant under the Nemenyi test. RF is therefore a suitable option under the tested conditions, though not necessarily optimal for all sEMG applications.

Synthetic-to-live degradation ranged from 27.5 to 40.5 pp (RDI 43.7–67.4%), with RF the most robust classifier. Held-out permutation importance identified WL, RMS and MAV as the most informative features. The CPI of the prototype is 13.77 using a dimensionless cost ratio and a USD 232 system cost. Above-chance three-class discrimination and light-object actuation were demonstrated, while amputee performance, long-term endurance and standalone operation remain to be established.

### Future work

Building upon the current findings, the following directions are proposed.

1. **Dual-channel acquisition:** Adding a second MyoWare sensor on the extensor compartment to improve G2 classification through antagonist muscle pair analysis.
2. **Transfer learning and domain adaptation:** Applying domain adaptation techniques (e.g., maximum mean discrepancy minimisation) to bridge the synthetic-to-real gap.
3. **Adaptive learning:** Implementing online incremental learning in the Random Forest to adapt to individual users' EMG patterns over time.
4. **Expanded gesture vocabulary:** Increasing to 5–8 gesture classes while maintaining acceptable accuracy.
5. **Embedded deployment:** Porting the RF model to a 32-bit MCU (e.g. ESP32 or ARM Cortex-M4) using tree pruning and TinyML quantization for fully standalone operation.
6. **Clinical validation:** Evaluating the system with a larger cohort including trans-radial amputee users in collaboration with rehabilitation clinicians.
7. **Sensor upgrade path:** Evaluating the MyoWare 2.0 sensor and other emerging low-cost sEMG platforms (e.g., OpenBCI Cyton) to quantify the performance improvement achievable with incremental hardware investment.
8. **Multi-session and longitudinal studies:** Assessing inter-session robustness and the long-term learning curve of users adapting to the myoelectric controller over weeks of daily use.
9. **Rejection thresholds and majority voting:** Implementing confidence-based gesture rejection and sliding-window majority voting to improve functional reliability in continuous real-time use.
10. **User experience evaluation:** Conducting standardised functional assessments (e.g., Southampton Hand Assessment Procedure, Box and Blocks Test) with both healthy subjects and amputee users to complement algorithmic metrics with clinically validated outcome measures.
